# Supplementary material for: Risk factors for recurrent endometriosis after conservative surgery in a quaternary care center in southern Thailand
Source: PLoS One. 2023 Aug 10;18(8):e0289832. doi: 10.1371/journal.pone.0289832 (PMC10414623; doi:10.1371/journal.pone.0289832)
Supplement: S1 Table — (DOCX) [file pone.0289832.s001.docx]

**S1 Table. Comparison of variables between non-recurrence and recurrence groups of conservative surgery in endometriosis**

|  | No recurrence | Recurrence | Total | Test stat. | P value |
| --- | --- | --- | --- | --- | --- |
| Number | 158 | 204 | 362 |  |  |
| Age at surgery (year) | | | | Ranksum test | 0.004 |
| median (IQR) | 35 (30.2,40) | 33 (29,37) | 34 (29,38) |  |  |
|  | | | | | |
| Religion | | | | Fisher's exact test | 0.364 |
| Buddhism | 137 (43.2) | 180 (56.8) | 317 (100) |  |  |
| Islam | 19 (44.2) | 24 (55.8) | 43 (100) |  |  |
| Christianity | 2 (100) | 0 (0) | 2 (100) |  |  |
|  | | | | | |
| Parity | | | | Ranksum test | 0.981 |
| median (IQR) | 0 (0,1) | 0 (0,1) | 0 (0,1) |  |  |
|  | | | | | |
| Abortion | | | | Ranksum test | 0.856 |
| median (IQR) | 0 (0,0) | 0 (0,0) | 0 (0,0) |  |  |
|  | | | | | |
| BMI (kg/m^2^) | | | | Ranksum test | 0.966 |
| median (IQR) | 21.4 (19.5,23.9) | 21.9 (19.2,23.8) | 21.7 (19.4,23.9) |  |  |
|  | | | | | |
| Previous abdominal surgery | | | | Chisq. (1 df) = 0.81 | 0.368 |
| Yes | 43 (48.3) | 46 (51.7) | 89 (100) |  |  |
| No | 115 (42.1) | 158 (57.9) | 273 (100) |  |  |
|  | | | | | |
| Sexual intercourse | | | | Chisq. (1 df) = 0 | 1 |
| No | 41 (44.1) | 52 (55.9) | 93 (100) |  |  |
| Yes | 117 (43.5) | 152 (56.5) | 269 (100) |  |  |
|  | | | | | |
| Primary dysmenorrhea | | |  | Chisq. (1 df) = 0.24 | 0.621 |
| No | 63 (45.7) | 75 (54.3) | 138 (100) |  |  |
| Yes | 95 (42.4) | 129 (57.6) | 224 (100) |  |  |
|  | | | | | |
| Secondary dysmenorrhea | | | | Chisq. (1 df) = 2.33 | 0.127 |
| No | 40 (51.9) | 37 (48.1) | 77 (100) |  |  |
| Yes | 118 (41.4) | 167 (58.6) | 285 (100) |  |  |
|  | | | | | |
| Duration from secondary dysmenorrhea (month) | | | | Ranksum test | < 0.001 |
| median (IQR) | 11 (5,24) | 24 (7.5,36) | 12 (6,36) |  |  |
|  | | | | | |
| Duration of secondary dysmenorrhea | | | | Chisq. (1 df) = 12.86 | < 0.001 |
| < 12 months | 61 (55) | 50 (45) | 111 (100) |  |  |
| ≥ 12 months | 57 (32.8) | 117 (67.2) | 174 (100) |  |  |
|  | | | | | |
| Chief complaint | | | | Chisq. (7 df) = 9.91 | 0.194 |
| Cyclic pain | 69 (39) | 108 (61) | 177 (100) |  |  |
| Acyclic pain | 25 (54.3) | 21 (45.7) | 46 (100) |  |  |
| Infertility | 12 (38.7) | 19 (61.3) | 31 (100) |  |  |
| Pelvic mass | 11 (39.3) | 17 (60.7) | 28 (100) |  |  |
| Abnormal uterine bleeding | 10 (43.5) | 13 (56.5) | 23 (100) |  |  |
| Hematochezia | 0 (0) | 2 (100) | 2 (100) |  |  |
| Accidental finding | 10 (50) | 10 (50) | 20 (100) |  |  |
| Acute pelvic pain | 21 (60) | 14 (40) | 35 (100) |  |  |
|  | | | | | |
| VAS score | | | | Ranksum test | 0.106 |
| median (IQR) | 6 (5,8) | 6 (5,9) | 6 (5,9) |  |  |
|  | | | | | |
| Severity of pelvic pain | | | | Chisq. (1 df) = 3.33 | 0.068 |
| none to mild | 50 (52.1) | 46 (47.9) | 96 (100) |  |  |
| moderate to severe | 108 (40.6) | 158 (59.4) | 266 (100) |  |  |
|  | | | | | |
| Cyclic pain | | | | Chisq. (1 df) = 1.77 | 0.184 |
| No | 42 (50.6) | 41 (49.4) | 83 (100) |  |  |
| Yes | 116 (41.6) | 163 (58.4) | 279 (100) |  |  |
|  | | | | | |
| Acyclic pain | | | | Chisq. (1 df) = 0 | 1 |
| No | 133 (43.8) | 171 (56.2) | 304 (100) |  |  |
| Yes | 25 (43.1) | 33 (56.9) | 58 (100) |  |  |
|  | | | | | |
| Dyspareunia | | | | Chisq. (1 df) = 2.39 | 0.122 |
| No | 146 (45.2) | 177 (54.8) | 323 (100) |  |  |
| Yes | 12 (30.8) | 27 (69.2) | 39 (100) |  |  |
|  | | | | | |
| Dyschezia | | | | Chisq. (1 df) = 0.22 | 0.636 |
| No | 145 (43.2) | 191 (56.8) | 336 (100) |  |  |
| Yes | 13 (50) | 13 (50) | 26 (100) |  |  |
|  | | | | | |
| Infertile | | | | Chisq. (1 df) = 8.16 | 0.004 |
| No | 120 (48.6) | 127 (51.4) | 247 (100) |  |  |
| Yes | 36 (31.9) | 77 (68.1) | 113 (100) |  |  |
|  | | | | | |
| Abnormal menstruation | | | | Chisq. (1 df) = 0.08 | 0.78 |
| No | 141 (43.3) | 185 (56.7) | 326 (100) |  |  |
| Yes | 17 (47.2) | 19 (52.8) | 36 (100) |  |  |
|  | | | | | |
| Hematochezia | | | | Fisher's exact test | 0.135 |
| No | 158 (44.1) | 200 (55.9) | 358 (100) |  |  |
| Yes | 0 (0) | 4 (100) | 4 (100) |  |  |
|  | | | | | |
| Accidental finding | | | | Chisq. (1 df) = 0.22 | 0.637 |
| No | 131 (43) | 174 (57) | 305 (100) |  |  |
| Yes | 27 (47.4) | 30 (52.6) | 57 (100) |  |  |
|  | | | | | |
| Palpation of pelvic mass | | | | Chisq. (1 df) = 8.95 | 0.003 |
| No | 104 (39.1) | 162 (60.9) | 266 (100) |  |  |
| Yes | 44 (59.5) | 30 (40.5) | 74 (100) |  |  |
|  | | | | | |
| Cul-de-sac nodularity | | | | Chisq. (1 df) = 0.01 | 0.929 |
| No | 38 (42.7) | 51 (57.3) | 89 (100) |  |  |
| Yes | 36 (40.9) | 52 (59.1) | 88 (100) |  |  |
|  | | | | | |
| Lateral or Cervical displacement | | | | Chisq. (1 df) = 0 | 1 |
| No | 12 (50) | 12 (50) | 24 (100) |  |  |
| Yes | 39 (48.8) | 41 (51.2) | 80 (100) |  |  |
|  | | | | | |
| Previous Hormonal treatment | | | | Chisq. (1 df) = 0.98 | 0.321 |
| No | 122 (45.4) | 147 (54.6) | 269 (100) |  |  |
| Yes | 36 (38.7) | 57 (61.3) | 93 (100) |  |  |
|  | | | | | |
| Duration of Hormonal treatment before surgery (month) | | | | Ranksum test | 0.766 |
| median (IQR) | 3 (2,3) | 3 (1.8,4.2) | 3 (2,3.5) |  |  |
|  | | | | | |
| Duration of Hormonal treatment before surgery | | | | Chisq. (1 df) = 0.78 | 0.376 |
| < 3 months | 11 (40.7) | 16 (59.3) | 27 (100) |  |  |
| ≥ 3 months | 24 (54.5) | 20 (45.5) | 44 (100) |  |  |
|  | | | | | |
| Duration between decision from diagnosis to surgery (month) | | | | Ranksum test | 0.675 |
| median (IQR) | 2 (1,3) | 2 (1,3) | 2 (1,3) |  |  |
|  | | | | | |
| Surgical approach | | | | Chisq. (1 df) = 6.48 | 0.011 |
| Laparoscopy | 105 (39.5) | 161 (60.5) | 266 (100) |  |  |
| Laparotomy | 53 (55.2) | 43 (44.8) | 96 (100) |  |  |
|  | | | | | |
| Conversion to laparotomy | | | | Chisq. (1 df) = 0.3 | 0.585 |
| No | 99 (39.9) | 149 (60.1) | 248 (100) |  |  |
| Yes | 6 (33.3) | 12 (66.7) | 18 (100) |  |  |
|  | | | | | |
| Operator | | | | Fisher's exact test | 0.124 |
| Staff | 153 (44.3) | 192 (55.7) | 345 (100) |  |  |
| Fellow | 5 (29.4) | 12 (70.6) | 17 (100) |  |  |
|  |  |  |  |  |  |
| Operative times (minutes) | | | | Ranksum test | 0.018 |
| median (IQR) | 150 (130,195) | 145 (115,175.5) | 150 (120,185) |  |  |
|  | | | | | |
| Operative procedure | | | | Fisher's exact test | < 0.001 |
| Adhesiolysis | 4 (20.0) | 16 (80.0) | 20 (100) |  |  |
| Ablation | 8 (24.2) | 25 (75.8) | 33 (100) |  |  |
| Ovarian cystectomy with or without adhesiolysis/ ablation/ cystectomy | 103 (41.9) | 143 (58.1) | 246 (100) |  |  |
| Unilateral oophorectomy with or without adhesiolysis/ ablation/ cystectomy | 42 (66.7) | 21 (33.3) | 63 (100) |  |  |
|  | | | | | |
| Obliterate cul-de-sac | | |  | Chisq. (2 df) = 1.86 | 0.394 |
| No | 32 (43.2) | 42 (56.8) | 74 (100) |  |  |
| Yes | 104 (45.8) | 123 (54.2) | 227 (100) |  |  |
| No data | 22 (36.1) | 39 (63.9) | 61 (100) |  |  |
|  | | | | | |
| Uterosacral ligament endometriosis | | |  | Chisq. (2 df) = 1.34 | 0.247 |
| No | 25 (49) | 26 (51) | 51 (100) |  |  |
| Yes | 21 (36.2) | 37 (63.8) | 58 (100) |  |  |
| No data | 112 (44.3) | 141 (55.7) | 253 (100) |  |  |
|  | | | | | |
| Deep infiltrative endometriosis | | | | Chisq. (2 df) = 0.16 | 0.693 |
| No | 25 (47.2) | 28 (52.8) | 53 (100) |  |  |
| Yes | 6 (37.5) | 10 (62.5) | 16 (100) |  |  |
| No data | 127 (44.3) | 166 (56.7) | 293 (100) |  |  |
|  | | | | | |
| Endometrioma | | | | Chisq. (1 df) = 11.3 | < 0.001 |
| No | 10 (20.8) | 38 (79.2) | 48 (100) |  |  |
| Yes | 148 (47.1) | 166 (52.9) | 314 (100) |  |  |
|  | | | | | |
| Size of largest endometrioma (mm) | | | | Ranksum test | 0.085 |
| median (IQR) | 60 (50,80) | 50 (40,80) | 50 (40,80) |  |  |
|  | | | | | |
| Side of endometrioma | | | | Chisq. (1 df) = 0.19 | 0.665 |
| Unilateral | 81 (42.9) | 108 (57.1) | 189 (100) |  |  |
| Bilateral | 57 (45.6) | 68 (54.4) | 125 (100) |  |  |
|  | | | | | |
| Pelvic endometriosis | | | | Chisq. (2 df) = 3.34 | 0.067 |
| No | 19 (59.4) | 13 (40.6) | 32 (100) |  |  |
| Yes | 56 (39.7) | 85 (60.3) | 141 (100) |  |  |
| No data | 83 (43.9) | 106 (56.1) | 189 (100) |  |  |
|  | | | | | |
| Co-diagnosis: Adenomyosis | | | | Chisq. (1 df) = 2.49 | 0.114 |
| No | 122 (41.5) | 172 (58.5) | 294 (100) |  |  |
| Yes | 36 (52.9) | 32 (47.1) | 68 (100) |  |  |
|  | | | | | |
| rAFS score | | | | Ranksum test | 0.895 |
| median (IQR) | 48 (28,87.5) | 60 (28,92) | 60 (28,89) |  |  |
|  | | | | | |
| Severity/ Stage of rAFS score | | | | Fisher's exact test | 0.017 |
| Minimal/ I | 4 (30.8) | 9 (69.2) | 13 (100) |  |  |
| Mild/ II | 1 (11.1) | 8 (88.9) | 9 (100) |  |  |
| Moderate/ III | 46 (56.1) | 36 (43.9) | 82 (100) |  |  |
| Severe/ IV | 75 (41.9) | 104 (58.1) | 179 (100) |  |  |
| No data | 32 (40.5) | 47 (59.5) | 79 (100) |  |  |
|  | | | | | |
| Complications | | | | Chisq. (1 df) = 0.68 | 0.411 |
| No | 139 (42.8) | 186 (57.2) | 325 (100) |  |  |
| Yes | 19 (51.4) | 18 (48.6) | 37 (100) |  |  |
|  | | | | | |
| Postoperative hormonal treatment | | | | Chisq. (1 df) = 19.99 | < 0.001 |
| No | 34 (27.2) | 91 (72.8) | 125 (100) |  |  |
| Yes | 124 (52.3) | 113 (47.7) | 237 (100) |  |  |
|  | | | | | |
| Time to receive hormonal treatment after surgery (month) | | |  | Ranksum test | 0.398 |
| median (IQR) | 3 (1,4) | 2 (0,4) | 2 (0.5,4) |  |  |
|  | | | | | |
| Duration of adjuvant treatment (month) | | | | Ranksum test | < 0.001 |
| median (IQR) | 31 (24,51) | 15 (8,27) | 26 (13,39.5) |  |  |
|  | | | | | |
| Type of postoperative hormonal treatment (some patients were received more than one treatment) | | | | | |
| *Cyclic Combined Pills* | | | | Chisq. (1 df) = 1.59 | 0.207 |
| No | 90 (47.9) | 98 (52.1) | 188 (100) |  |  |
| Yes | 18 (36.7) | 31 (63.3) | 49 (100) |  |  |
| *Continued Combined Pills* | | | | Chisq. (1 df) = 9.71 | 0.002 |
| No | 75 (41.9) | 104 (58.1) | 179 (100) |  |  |
| Yes | 38 (65.5) | 20 (34.5) | 58 (100) |  |  |
| *Dienogest* | | | | Chisq. (1 df) = 2.33 | 0.127 |
| No | 88 (44.4) | 110 (55.6) | 198 (100) |  |  |
| Yes | 23 (59) | 16 (41) | 39 (100) |  |  |
| *Monthly injectable contraceptives* | | | | Fisher's exact test | 0.184 |
| No | 107 (46.9) | 121 (53.1) | 228 (100) |  |  |
| Yes | 2 (22.2) | 7 (77.8) | 9 (100) |  |  |
| *Depot medroxyprogesterone acetate* | | | | Chisq. (1 df) = 10.74 | 0.001 |
| No | 30 (36.1) | 53 (63.9) | 83 (100) |  |  |
| Yes | 86 (55.8) | 68 (44.2) | 154 (100) |  |  |
| *Levonorgestrel releasing intrauterine system* | | | | Chisq. (1 df) = 2.19 | 0.139 |
| No | 103 (45.6) | 123 (54.4) | 226 (100) |  |  |
| Yes | 8 (72.7) | 3 (27.3) | 11 (100) |  |  |
| *Gonadotropin releasing hormone agonists* | | | | Chisq. (1 df) = 1.41 | 0.234 |
| No | 84 (44.7) | 104 (55.3) | 188 (100) |  |  |
| Yes | 27 (55.1) | 22 (44.9) | 49 (100) |  |  |
|  | | | | | |
| Duration of follow-up (month) | | | | Ranksum test | < 0.001 |
| median (IQR) | 43.5 (29,63) | 68 (37.8,100.2) | 53 (31,88) |  |  |

BMI, body mass index; IQR, interquartile range; mm, millimeter; rAFS, revised American Fertility Society; VAS, visual analog scale.

*P* values of 0.05 were significant and analyzed between the non-recurrence and recurrence groups.
